# Ion Mobility Mass Spectrometry Reveals Rare Sialylated Glycosphingolipid Structures in Human Cerebrospinal Fluid

**DOI:** 10.3390/molecules27030743

**Published:** 2022-01-24

**Authors:** Mirela Sarbu, Dragana Fabris, Željka Vukelić, David E. Clemmer, Alina D. Zamfir

**Affiliations:** 1 Department of Condensed Matter, National Institute for Research and Development in Electrochemistry and Condensed Matter, 300569 Timisoara, Romania; mirela.sarbu86@yahoo.co.uk; 2 Department of Physics, West University of Timisoara, 300223 Timisoara, Romania; 3 Department of Chemistry and Biochemistry, School of Medicine, University of Zagreb, 10000 Zagreb, Croatia; dragana.fabris@gmail.com (D.F.); zeljka.vukelic@mef.hr (Ž.V.); 4 Department of Chemistry, Indiana University, Bloomington, IN 47405, USA; clemmer@indiana.edu; 5 Department of Technical and Natural Sciences, ‘‘Aurel Vlaicu’’ University of Arad, 310330 Arad, Romania

**Keywords:** ion mobility separation mass spectrometry (IMS MS), sialylated glycosphingolipids, gangliosides, human cerebrospinal fluid, collision-induced dissociation, GalNAc-GD1c isomer

## Abstract

Gangliosides (GGs) represent an important class of biomolecules associated with the central nervous system (CNS). In view of their special role at a CNS level, GGs are valuable diagnostic markers and prospective therapeutic agents. By ion mobility separation mass spectrometry (IMS MS), recently implemented by us in the investigation of human CNS gangliosidome, we previously discovered a similarity between GG profiles in CSF and the brain. Based on these findings, we developed IMS tandem MS (MS/MS) to characterize rare human CSF glycoforms, with a potential biomarker role. To investigate the oligosaccharide and ceramide structures, the ions detected following IMS MS separation were submitted to structural analysis by collision-induced dissociation (CID) MS/MS in the transfer cell. The IMS evidence on only one mobility feature, together with the diagnostic fragment ions, allowed the unequivocal identification of isomers in the CSF. Hence, by IMS MS/MS, GalNAc-GD1c(d18:1/18:1) and GalNAc-GD1c(d18:1/18:0) having both Neu5Ac residues and GalNAc attached to the external galactose were for the first time discovered and structurally characterized. The present results demonstrate the high potential of IMS MS/MS for biomarker discovery and characterization in body fluids, and the perspectives of method implementation in clinical analyses targeting the early diagnosis of CNS diseases through molecular fingerprints.

## 1. Introduction

Glycosphingolipids (GSLs), comprising a ceramide backbone linked by a β-glycosidic bond to complex glycans, are located in the outer leaflet of the mammals, plants and bacteria plasma membrane [1]. The central nervous system (CNS) is abundant in acidic GSLs, such as sialic acid-containing GSLs (gangliosides, GGs) and sulfate-containing GSLs (sulfoglycolipids) [2]. These two classes of molecules are involved in neurodevelopment, neural cell recognition and cell–cell adhesion [1]. As constituents of cell membrane microdomains, GGs contribute to extracellular biologic events, such as the modulation of membrane proteins and ion channels, cell–cell recognition, adhesion, cellular differentiation, growth and intra- and intercellular signaling [3,4,5]. Loss-of-function mutations in GG biosynthetic enzymes cause severe neurodegenerative disorders [3,6,7,8,9]; alterations in the composition and concentration of particular GGs might also occur with aging [10,11,12] and in common neurodegenerative conditions, such as Huntington’s disease, Alzheimer’s disease, Parkinson’s disease, amyotrophic lateral sclerosis, stroke, multiple sclerosis and epilepsy [13,14]. Therefore, GGs are not only markers for cells at certain developmental stages, but also prone to become targets of disease-associated antibodies [1].

Cerebrospinal fluid (CSF), a clear, colorless plasma-like fluid, located within the subarachnoid space that bathes the CNS and can be collected through lumbar puncture, represents nowadays a body fluid whose examination gained more interest for clinical diagnosis of a wide range of CNS diseases that otherwise would be difficult to diagnose. Since, in adults, there is a daily secretion of 400–600 mL of CSF with an unchanged composition, which upholds a stable intraventricular environment in the brain, essential for maintaining normal neural functions [15], CSF is renewed four to five times daily. Hence, any disease of CNS leads to alterations in the normal appearance and composition of the CSF. Autoimmune diseases [16,17,18], primary or metastatic cancers [19,20,21], any hemorrhage in or around the brain [22], Alzheimer’s disease [23,24,25] and infectious diseases, such as meningitis [26] and encephalitis [27,28], are some of the CNS conditions that can be diagnosed by the appearance, cytology and evaluation of the level or concentration of different components in CSF.

Whereas GGs are released into the intercellular space through the normal cell-membrane turnover, known as “cell surface shedding” [29], and the intercellular space in the brain communicates with the CSF, the GG rate of release is reflected in the concentration of GGs in the CSF [30,31]. Furthermore, altered concentrations of GGs in the CSF could reflect an abnormal nerve-cell turnover, including the abnormal turnover of synapses [32].

**Scheme 1:**
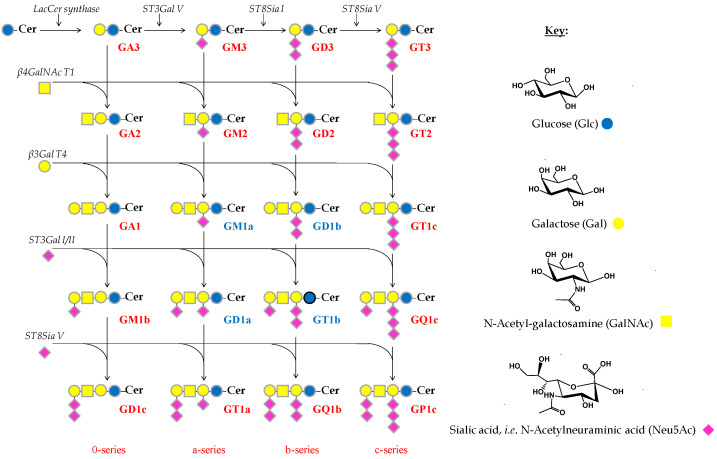
Biosynthesis pathway of the main brain gangliosides.

Although CSF sampling is more difficult than urine or blood, as CSF is in direct contact with the brain and spine, CSF testing is more effective in diagnosing a variety of CNS conditions. While the human brain, in particular the cerebral cortex, is characterized by a GG concentration of about 3000–3500 nM NeuAc/g of fresh tissue [3], the GG content in CSF is greatly reduced, up to only 0.3–0.5 nM NeuAc/mL [31,33] and an average of 92 nM of the four main brain GGs (GM1, GD1a, GD1b and GT1b) (Scheme 1) [34]. Thus, the extraction, quantification and analysis of GGs in CSF require high performance techniques, characterized by elevated sensitivity and resolution.

Ion mobility separation mass spectrometry (IMS MS) has emerged as a powerful and ultrafast technique, able to provide an excellent and reproducible separation based on the dissimilarities in the collision cross section and charge as the ions pass through a buffer gas under an electric field [35,36,37,38]. In recent years, the method has gained in popularity in the glycolipidomic field and related clinical assays [39,40,41], mainly because IMS (i) is not a stand-alone instrument, being incorporated within the MS instrument; (ii) does not imply special solvents or complicated sample preparation procedures prior to mass analysis; (iii) allows the real-time separations and characterization of highly complex mixtures; (iv) offers information of possible glycolipid isomers, isobars and conformers in biological mixture; (v) allows the detection and identification of low abundant ions, indistinguishable solely by MS; and vi) can function properly for high-throughput analyses.

In glycosphingolipidomics, the exhaustive separation of the molecules according to the mass-to-charge ratio, charge state, carbohydrate chain length, the sialylation degree and ceramide composition, as revealed by the published IMS MS literature [11,42,43,44,45], allowed the identification of novel structures, characterized by a higher degree of sialylation and diversity of the ceramide composition. Recently, IMS MS implemented by us in the investigation of human CSF gangliosidome in a pilot study [46], offered a first overview on the GG pattern and highlighted the incidence of a similar GG profile in human CSF and brain. Given the valuable data provided by IMS MS for CSF, a detailed evaluation of the GG profile in CSF revealed the presence of new and biologically relevant species. Hence, in the current study, we explored the incidence of potential biomarkers belonging to the disialoganglioside class with the longest glycan chain (GD1) and exhibiting elongated structures present in the normal lumbar human CSF. Such species, previously detected in the human brain and serum [47,48,49,50,51,52,53,54], were not structurally investigated. The prevalence of GalNAc-GD1 glycoforms in CSF, thoroughly characterized by collision-induced dissociation (CID) MS/MS, documents the shedding of glycolipids from the brain into CSF.

In view of the (i) permanent renewal of CSF; (ii) limited access to brain biopsies or tumor tissue samples; (iii) vital role played by GGs in neurodevelopment; (iv) drastically reduced GG content in CSF compared to human brain; (v) previous observations related to the similarity of GG content in CSF and brain and (vi) detection, identification and structural characterization by IMS CID MS/MS of biologically relevant molecules of lower expression, IMS MS approach generated here data useful for early diagnosis of CNS conditions. Based on the discovered GG molecular fingerprints, a systematic assessment of either biochemical changes occurring with disease progression/regression or the follow-up treatment might also be possible.

## 2. Results

### 2.1. IMS MS Screening

Ten μL of purified native CSF ganglioside mixture dissolved in methanol at a concentration of 5 pmol/µL was infused into Synapt G2S and analyzed in negative ion mode nanoESI IMS MS. As previously reported for this type of molecule [11,42,43,46], IMS separates on a millisecond time-scale the GG species into mobility groups, based on the charge state, carbohydrate and ceramide chain lengths and degree of sialylation. This is a major advantage, as it allows (i) the detection of a larger number of species, including those of low abundance, which could not be detected based solely on *m*/*z* separation, and (ii) the extraction and processing only of the data of interest, such as the GD1 species investigated here in more detail, by retaining the relevant drift time and combining the chromatogram.

**Figure 1:**
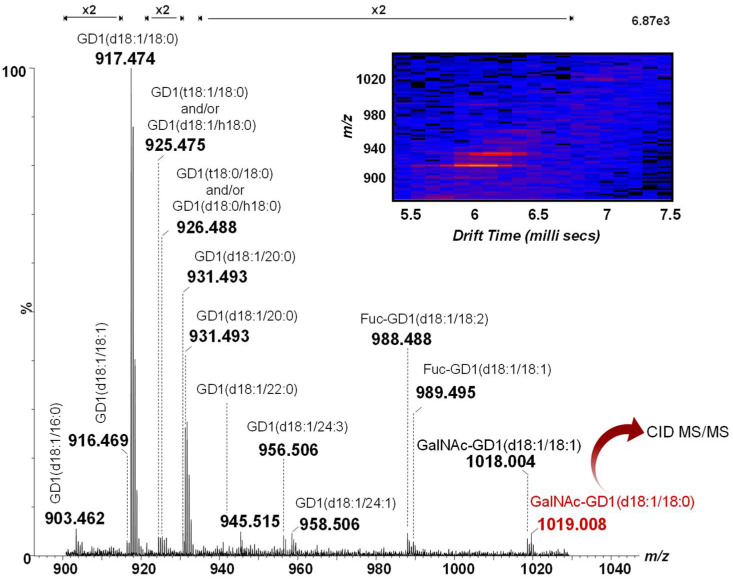
Combined (-) nanoESI IMS MS of doubly deprotonated GD1 species from the extracted drift time corresponding to the GD1 class in the CSF sample. *Inset*: Driftscope display (drift time vs. *m*/*z*) of the doubly charged GD1 class.

Hence, the mass spectrum and the 2D DriftScope plot (*m*/*z* vs. drift time) of the doubly charged GD1 class in methanol, generated after 2 min of signal acquisition, is depicted in Figure 1. A closer inspection of Figure 1 reveals the incidence of 14 different GD1 species. As observed from both the MS and the DriftScope presented as an inset in Figure 1, the most abundant ions at *m*/*z* 917.474 and *m*/*z* 931.493 correspond to two species encompassing the most common ceramides: GD1(d18:1/18:0) and GD1(d18:1/20:0), respectively. However, fatty acids with chain lengths from 16 up to 24 carbon atoms in their composition were observed as well. The ion at *m*/*z* 925.475, shifted with 16 amu with respect to the base peak, could arise from either the GD1(t18:1/18:0) or the GD1(d18:1/h18:0) molecular species. In addition to the diversity of the ceramide chains, the prevalence of biologically-relevant modifications, such as *O*-fucosylation, commonly found in the human brain [11,42,43], was observed as well. Fuc-GD1(d18:1/18:2) and Fuc-GD1(d18:1/18:1) previously detected both in the human brain [11,12,54] and CSF [46] were found here at *m*/*z* 988.488 and *m*/*z* 989.495, respectively, at sufficient abundances for their straightforward assignment.

Of particular importance are the doubly charged ions at *m*/*z* 1018.004 and *m*/*z* 1019.008 assigned, according to mass calculation, to GalNAc-GD1(d18:1/18:1) and GalNAc-GD1(d18:1/18:0). Whereas the GalNAc-GD1a isomer was associated with the pure motor variant of Guillain–Barré syndrome [47,48,49,50], GalNAc-GD1c-like lipo-oligosaccharides were detected in *C. jejuni* isolates from Fisher syndrome patients one decade ago [51], but not associated with the disorder. SinceMS^1^ was unable to reveal data related to the molecular structure, the ion at *m*/*z* 1019.008 was selected and submitted to further structural investigation using collision-induced dissociation at low collision energies in order to elucidate its molecular architecture.

### 2.2. Structural Elucidation by IMS CID MS/MS of Uncommon Glycoforms

**Scheme 2:**
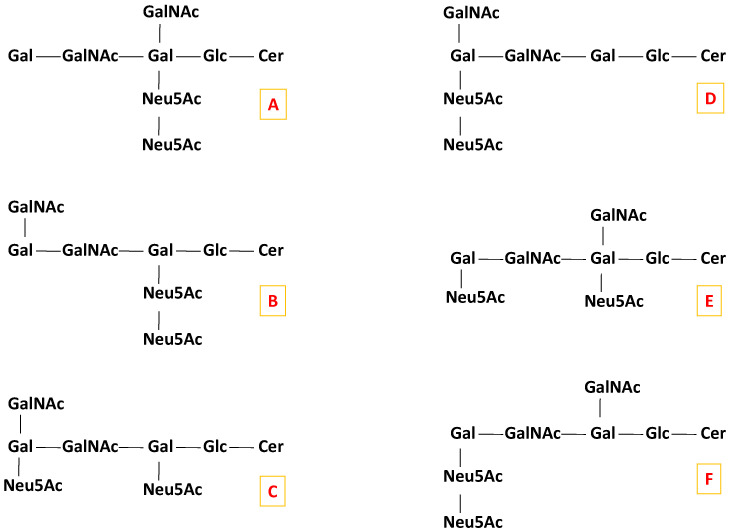
Possible candidate isomers for the GalNAc-GD1(d18:1/18:0) molecular form.

IMS MS demonstrated the occurrence in CSF of elongated glycoforms, such as GalNAc-GD1, a minor GG specific to the human brain and peripheral nerve [49,52,53]. Such an example is the ion detected at *m*/*z* 1019.006 in Figure 1 and assigned, according to mass calculation, to the doubly deprotonated GalNAc-GD1(d18:1/18:0). This species was previously found in normal human adult cerebellum tissue [54] using automated chip-based nanoelectrospray high resolution MS, but not structurally characterized. Hence, we have isolated the ion by setting LM and HM to 15, and submitted it to low energy CID MS/MS in the negative ion mode for a detailed investigation of the oligosaccharide and ceramide structures. Data upon the localization of Neu5Ac residues essentially for isomer identification from the six possible structures illustrated in Scheme 2 that might be present in CSF were collected as well.

**Figure 2:**
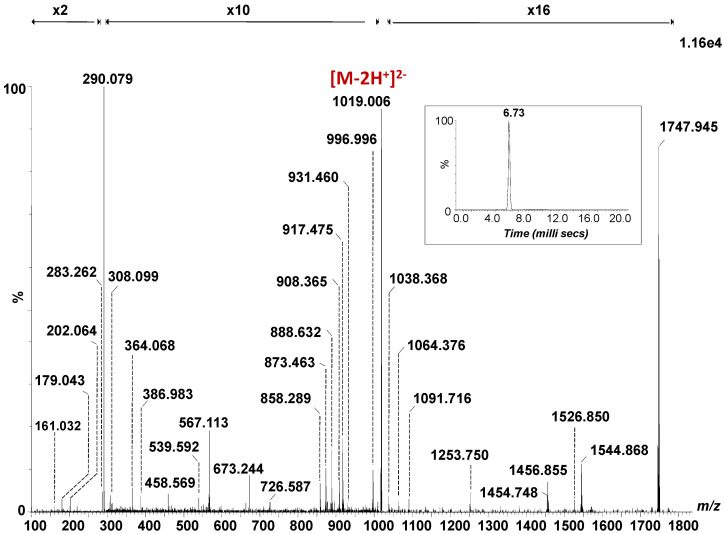
(-) nanoESI IMS CID MS/MS of the [M − 2H]^2-^ at *m*/*z* 1019.006 corresponding to the GalNAc-GD1(d18:1/18:0) species isolated and fragmented from CSF sample. Inset: drift time distribution for the ion at *m*/*z* 1019.006 fragmented by CID. Cone voltage 45 V. Capillary voltage 1.6 kV. Acquisition 180 scans. CID at variable collision energy within 30–40 eV.

The fragmentation spectrum of the [M − 2H]^2-^ detected at *m*/*z* 1019.006, generated by combining the total ion chromatogram, acquired for 3 min under variable collision energy within 30–40 eV range, is depicted in Figure 2, together with its single mobility feature at 6.73 ms (Inset Figure 2), which substantiates the occurrence of a single isomer. The assignment of the detected fragment ions in the spectrum in Figure 2 is presented in Table 1.

Considering the isolation window of one *m*/*z* unit for this parent ion, the fragment ions of the GalNAc-GD1(d18:1/18:1) species, with an additional double bond in the composition of the fatty acid, detected at *m*/*z* 1018.004 in Figure 1, selected and fragmented in parallel with its analogue, were also observed and designated with asterisk in Table 1.

The abundant B_1α_ and C_1α_ ions detected at *m*/*z* 290.079 and *m*/*z* 308.099 generated following the detachment of one Neu5Ac residue from the parent ion, document the sialylation status of the molecule. B_1β_ or Y_3_/Y_2_ at *m*/*z* 202.064 and C_1β_ or Y_3_/Z_2_ at *m*/*z* 220.072, respectively, are diagnostic for the GalNAc fragment. The following sequence ions detected as B_3_/B_2α_, B_4_/B_2α_, C_3_/B_1α_ or Y_4β_/B_1α_/Z_2_, B_4_/B_1α_ and C_4_/B_1α_ at *m*/*z* 364.068, *m*/*z* 567.113, *m*/*z* 673.244, *m*/*z* 858.289 and *m*/*z* 876.308, respectively, along with the above-mentioned fragment ions document together the structure of the non-reducing end.

On the other hand, Y_0_ at *m*/*z* 564.533 together with Z_0_ at *m*/*z* 546.541 and U at *m*/*z* 283.262 corresponding to the C18:0 fatty acid, validate (d18:1/18:0) ceramide composition, while Y_0_* at *m*/*z* 562.522 and U* at *m*/*z* 281.243 are indicative ofthe C18:1 fatty acid belonging to the GalNAc-GD1(d18:1/18:1) species, selected and fragmented simultaneously.

The fragment ions produced by the loss of one or both Neu5Ac and/or GalNAc residues from the precursor ions were also detected in the mass spectrum in Figure 2. Hence, the ions Y_5α_ at *m*/*z* 873.463 and *m*/*z* 1747.945; Y_4α_ at *m*/*z* 1456.855; Z_4β_ at *m*/*z* 908.365; Y_4β_ at *m*/*z* 917.475; Y_4β_/C_1α_ at *m*/*z* 1526.850; Y_4β_/B_1α_ at *m*/*z* 1544.868 and Y_4β_/B_2α_ at *m*/*z* 1253.750 are specific to the structure encompassing the (d18:1/18:0) ceramide composition. The ions at *m*/*z* 1745.889, *m*/*z* 1454.748, *m*/*z* 907.339, *m*/*z* 916.466 and *m*/*z* 1542.848, assigned to Y_5α_*, Y_4α_*, Z_4β_*, Y_4β_* and Y_4β_*/B_1α_ are diagnostic for the species with an additional double bond in the composition of the fatty acid chain.

Considering the Gal-GalNAc-Gal chain symmetry of the sugar core, and the possibility of the additional GalNAc and Neu5Ac residue attachment at any of the two Gal units, there are, theoretically, six possible structural candidates for the GalNAc-GD1(d18:1/18:0) formula, as illustrated in Scheme 2. The ion at *m*/*z* 552.594 formed by double bond and internal cross-ring cleavages and assigned to B_3_/B_1α_/^2,5^A_1β_ might arise only from the first five (A–E) candidates depicted in Scheme 2. Moreover, the ions at *m*/*z* 858.289 and *m*/*z* 876.308, corresponding to the Neu5Ac-(GalNAc)-Gal-GalNAc sequence are possible only for the (A), (C), (D) and (E) candidates. Consequently, only these four possible structural candidates from Scheme 2 were further considered.

**Figure 3:**
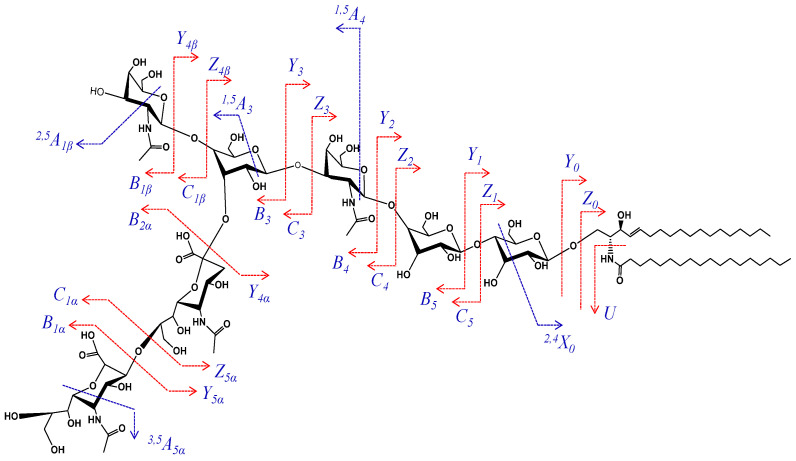
Fragmentation scheme of GalNAc-GD1(d18:1/18:0).

According to mass calculation, the ion at *m*/*z* 458.569 arising upon the cross-ring cleavage of a Gal unit and corresponding to the Neu5Ac_2_-GalNAc-Gal structure is a fragment ion supporting the incidence of the disialo element. More importantly, in view of the GalNAc (β1-4) Gal, GalNAc (β1-3) Gal or GalNAc (β1-6) Gal linkage possibilities, the fragment ions at *m*/*z* 539.592 and *m*/*z* 1064.376 assigned to ^1,5^A_4_/B_2α_ and C_4_/^2,5^A_1β_, together with the previously mentioned ones, are relevant for the attachment of the disialo element and the additional GalNAc residue to the external Gal residue. Therefore, these fragment ions support a structural motif consistent with the (D) candidate. The scheme depicting the fragmentation pathway experienced by the precursor ion during the dissociation event is provided in Figure 3.

Consequently, GalNAc-GD1c(d18:1/18:1) and GalNAc-GD1c(d18:1/18:0) are uncommon isomers, having both sialic acids and GalNAc attached to the external galactose unit were for the first time evidenced here in CSF and structurally characterized in detail.

## 3. Materials and Methods

### 3.1. Cerebrospinal Fluid Sampling

The investigation of lumbar CSF, sampled from adults exhibiting no signs of tumors, demyelination, intracranial hemorrhage, congenital errors of lipid metabolism or acute inflammatory process of the CNS, was carried out in order to exclude meningitis associated with the patients, through a meningitis epidemic episode. The lumbar puncture was not performed solely for ganglioside research. Approximately 1 mL of CSF was collected as a residual volume after clinical analysis from each individual patient diagnosed as healthy. A total of 21 mL of CSF was pooled and provided for scientific purposes. The CSFs were sampled by the Clinical Hospital Centre Zagreb, Department for General Clinical Biochemistry and Diagnostics of CSF, Croatia. Permission for the experiments with human material for scientific purposes was obtained from the Ethical Commission of the University of Zagreb, Croatia, under project no. 108120 by the Ministry of Science and Technology of the Republic of Croatia. All procedures on the human tissue and/or human body fluids were in agreement with the 1964 Helsinki Declaration and its later amendments. Subsequent to the biochemical examinations and prior ganglioside extraction, a CSF sample was centrifuged for 10 min at 6000 rpm in a mini-centrifuge (Thermo Fisher Scientific, U.S.A.) and the supernatant was collected.

### 3.2. Gangliosides Extraction and Purification

The extraction and purification of the CSF GG mixture followed the method developed by Svennerholm and Fredman [55], as modified by Vukelić et al. [56] and the procedures previously described [46]. Briefly, the CSF sample was treated as a corresponding water homogenate in the extraction procedure. Lipid extraction was performed twice using a chloroform–methanol–water mixture (1:2:0.75, by vol.). In order to separate the GGs from the other lipids, a phase partition followed by repartition was carried out by adding to the combined supernatant resulted after centrifugation of the chloroform, methanol and water up to a final volume ratio of 1:1:0.8. The combined upper phases containing polar glycosphingolipids (gangliosides) were collected. The crude GG extract was purified in several steps, as previously described by us [46]. The GG extract was dissolved in pure methanol and an aliquot was diluted in methanol to prepare a stock solution of 0.5 µg/mL (corresponding to approximately 700 µL of CSF). Prior to the experiments, the stock solution was stored at −40 °C.

### 3.3. Ion Mobility Mass Spectrometry

A Synapt G2S mass spectrometer (Waters, Manchester, U.K.) equipped with a nanoESI source was used for conducting the ion mobility mass spectrometry experiments in negative ion mode. The instrument was interfaced to a PC computer running the Waters MassLynx (version V4.1, SCN 855) and Waters Driftscope (version V2.7) software for data acquisition and IMS data processing.

The 10 cm long capillaries of uncoated borosilicate glass (ID: 1.2 mm, OD: 1.5 mm; Sutter Instrument Co., Novato, CA) were pulled with a Sutter p-97 micropipette puller to produce electrospray capillaries with 10 µm tip sizes and taper lengths of 4 mm. Ten µL of the CSF solution in methanol at 5 pmol/µL GG concentration was introduced into the back of a pulled emitter and a 0.25 mm platinum wire was inserted into the solution. All mass spectra (MS and MS/MS) were acquired in the negative ion mode, within the mass range of 100–2500 *m*/*z*, with a speed of 1 scan/s. The voltage applied to the platinum wire and the cone was carefully tuned to attain an efficient ionization of the components and a reduced in-source fragmentation. Hence, a constant spray was obtained at 1.6 kV and 45 V potential for the capillary and cone, respectively, while other parameters were set as follows: source block temperature 100 °C, desolvation gas flow rate 800 L/h and desolvation temperature 150°C. The low-mass (LM) and high-mass (HM) resolution parameters were set at 18 and 16, respectively, for the MS experiments, while for the CID MS/MS experiments, both were set at 15. To enhance the GG separation, the IMS parameters were thoroughly optimized as follows: wave velocity at 650 m/s, IMS wave height at 40 V and IMS gas flow at 90 L/min. These values provided the greatest separation of both the molecular ions in the mixture screened in the MS mode, and the mass-selected ions in the quadrupole submitted further to the CID MS/MS. The fragmentation experiments, using collision energies between 30 and 40 eV, were carried out in the transfer cell, placed after the mobility cell, as the discrimination of the parent ion isomers, if present, was provided.

### 3.4. Ganglioside Abbreviation and Assignment of the Spectra

The abbreviation of II^3^-α-(Neu5Ac)_2_-Gg_4_Cer as GD1 was according to the system of Svennerholm [57] and the recommendations of the IUPAC-IUB Commission on Biochemical Nomenclature (IUPAC-IUB 1998) [58]. The assignment of oligosaccharide sequence ions generated following the fragmentation experiment followed the generally accepted nomenclature introduced by Domon and Costello [59] and revised by Costello et al. [60]. Ceramide fragment ions were assigned according to the nomenclature of Ann and Adams [61] and based on the previously acquired knowledge on the structure of GGs in human CNS [44,46,62,63,64,65].

## 4. Conclusions

The results of the current study illustrate that ion mobility separation mass spectrometry is a powerful and extremely proficient technique capable of unambiguously identifying and structurally characterizing GG species having a low expression in the human CSF, but with a potential biomarker role. Ganglioside separation, according to their mobility through a buffer gas, directly influenced by the charge state, *m*/*z* ratio, carbohydrate and ceramide chain lengths and degree of sialylation, allowed for the identification for the first time in human CSF of rare structures exhibiting elongated glycan chains. The possibility to extract and retain the drift times for narrow regions of the 2D plot corresponding to the areas of interest also significantly contributed to the comprehensive mapping of the GGs in CSF. GalNAc-GD1(d18:1/18:0), a newly discovered species in CSF, detected as a low intensity ion, was submitted to structural characterization by CID MS/MS.

Each sequencing method applied to the structural analysis of glycans and glycoconjugates, whether it is the fragmentation of their native or permethylated forms by MS^n^, the digestion of the glycan chain by the use of glycosidases or the tandem MS performed after a proficient separation technique, exhibits both advantages and disadvantages. For certain studies, some methods are more suitable than others. The aim of the present work was to discover and characterize rare GG species in human CSF and highlight the major benefits for this purpose of a rigorously optimized IMS MS/MS approach.

This work emphasized not only the data consistency but also the analysis speed and the high reproducibility and sensitivity provided by IMS MS/MS. The latter parameters are crucial in such studies since clinical extracts are only available for research in limited amounts. Thus, for an identical instrumental setup and tuning, the current approach, based on a single run and on a single instrument, provided 100% in-run, about 98% run-to-run and 95% day-to-day reproducibility of the results, for a number of three replicates. In terms of sensitivity, for the sample concentration of 5 pmol/μL, 2 min signal acquisition for IMS screening and 3 min for structural characterization by CID MS/MS, are equivalent with a consumption of 2 μL for the entire study, which correspond to only 10 pmols of CSF GGs.

Unlike the methods based on the enzymatic fragmentation of the glycan chain, no post-digestion purification steps, which often result in potential sample loss, were necessary. Through the fragment ions generated by the cleavage of the glycosidic bonds and the cross-ring cleavages, IMS CID MS/MS can offer the information necessary to deduce the structure of the oligosaccharide chain and the ceramide for single GG components in complex mixtures.

While the incidence of a single mobility feature for the parent ion disclosed the existence of a single structural conformation, the optimized fragmentation conditions induced efficient ion dissociation with high sequence coverage. No less than 37 diagnostic fragment ions for GalNAc-GD1(d18:1/18:0) were generated, of which, 6 served for the unequivocal determination of the isomers present in CSF. Hence, GalNAc-GD1c(d18:1/18:1) and GalNAc-GD1c(d18:1/18:0) having both sialic acids and GalNAc attached to the external galactose unit were for the first time found in CSF and structurally characterized.

The current data substantiate the high efficiency of IMS MS and CID MS/MS techniques in revealing rare GG components and deciphering structural isomers in body fluids, where such species have a few times lower expression than in neural tissue. Such findings are of primary importance in relation to the future applicability of IMS MS in early diagnostic of CNS diseases, based on the discovery of biomarkers in CSF or blood/serum.

## Figures and Tables

**Table 1:** Assignment of the fragment ions originating from *m*/*z* 1019.006 and *m*/*z* 1018.002 corresponding to the GalNAc-GD1(d18:1/18:0) and GalNAc-GD1(d18:1/18:1) (denoted by asterisk *) species isolated from the CSF sample and fragmented by (-) nanoESI IMS CID MS/MS.

*m*/*z*	Charge State	Type of Fragment Ion
161.032	1-	Y_4β_/Y_3_/B_2α_ or Y_2_/Y_1_ or Y_1_/Y_0_
179.043	1-	Y_4β_/Z_3_/B_2α_ or Y_2_/Z_1_ or Y_1_/Z_0_
202.064	1-	B_1β_ or Y_3_/Y_2_
220.072	1-	C_1β_ or Y_3_/Z_2_
281.243	1-	U*
283.262	1-	U
290.079	1-	B_1α_
308.099	1-	C_1α_
364.068	1-	B_3_/ B_2α_
386.983	1-	^2,4^X_0_/U
458.569	2-	^1,5^A_3_
539.592	1-	^1,5^A_4_/B_2α_
546.541	1-	Z_0_
552.594	1-	B_3_/B_1α_/^2,5^A_1β_
562.522	1-	Y_0_*
564.533	1-	Y_0_
567.113	1-	B_4_/B_2α_
673.244	1-	C_3_/B_1α_ or Y_4β_/B_1α_/Z_2_
708.496	1-	Z_1_
724.578	1-	Y_1_*
726.587	1-	Y_1_
858.289	1-	B_4_/B_1α_
873.463	2-	Y_5α_
876.308	1-	C_4_/B_1α_
888.632	1-	Y_2_
895.480	2-	Y_4β_/CH_3_CHO
907.339	2-	Z_4β_*
908.365	2-	Z_4β_
916.466	2-	Y_4β_*
917.475	2-	Y_4β_
931.460	2-	^3,5^A_1α_
995.972	2-	M/*CH_3_CHO
996.996	2-	M/CH_3_CHO
1018.002	2-	[M* − 2H]^2-^
1019.006	2-	[M − 2H]^2-^
1038.368	1-	C_5_/B_1α_
1064.376	1-	C_4_/^2,5^A_1β_
1089.707	1-	Y_3_*
1091.716	1-	Y_3_
1253.750	1-	Y_4β_/B_2α_
1454.748	1-	Y_4α_*
1456.855	1-	Y_4α_
1526.850	1-	Y_4β_/C_1α_
1542.848	1-	Y_4β_*/B_1α_
1544.868	1-	Y_4β_/B_1α_
1745.889	1-	Y_5α_*
1747.945	1-	Y_5α_

## References

[1] Yu R., Yanagisawa M., Ariga T., Kamerling H. (2007). Glycosphingolipid Structures. Comprehensive Glycoscience.

[2] Ariga T., Yu R.K. (2005). Antiglycolipid antibodies in Guillain-Barré syndrome and related diseases: Review of clinical features and antibody specificities. J. Neurosci. Res..

[3] Sipione S., Monyror J., Galleguillos D., Steinberg N., Kadam V. (2020). Gangliosides in the Brain: Physiology, Pathophysiology and Therapeutic Applications. Front. Neurosci..

[4] Iwabuchi K., Yamamura S., Prinetti A., Handa K., Hakomori S.-I. (1998). GM3-enriched Microdomain Involved in Cell Adhesion and Signal Transduction through Carbohydrate-Carbohydrate Interaction in Mouse Melanoma B16 Cells. J. Biol. Chem..

[5] Yu R.K., Bieberich E., Xia T., Zeng G. (2004). Regulation of ganglioside biosynthesis in the nervous system. J. Lipid Res..

[6] Simpson M.A., Cross H., Proukakis C., Priestman D.A., Neville D.C.A., Reinkensmeier G., Wang H., Wiznitzer M., Gurtz K., Verganelaki A. (2004). Infantile-onset symptomatic epilepsy syndrome caused by a homozygous loss-of-function mutation of GM3 synthase. Nat. Genet..

[7] Boukhris A., Schüle-Freyer R., Loureiro J.L., Lourenço C.M., Mundwiller E., Gonzalez M.A., Charles P., Gauthier J., Rekik I., Lebrigio R.F.A. (2013). Alteration of Ganglioside Biosynthesis Responsible for Complex Hereditary Spastic Paraplegia. Am. J. Hum. Genet..

[8] Fragaki K., Ait-El-Mkadem S., Chaussenot A., Gire C., Mengual R., Bonesso L., Bénéteau M., Ricci J.E., Desquiret-Dumas V., Procaccio V. (2012). Refractory epilepsy and mitochondrial dysfunction due to GM3 synthase deficiency. Eur. J. Hum. Genet..

[9] Harlalka G.V., Lehman A., Chioza B., Baple E.L., Maroofian R., Cross H., Sreekantan-Nair A., Priestman D.A., Al-Turki S., McEntagart M.E. (2013). Mutations in B4GALNT1 (GM2 synthase) underlie a new disorder of ganglioside biosynthesis. Brain.

[10] Sarbu M., Dehelean L., Munteanu C., Vukelić Ž., Zamfir A.D. (2017). Assessment of ganglioside age-related and topographic specificity in human brain by Orbitrap mass spectrometry. Anal. Biochem..

[11] Sarbu M., Robu A.C., Ghiulai R.M., Vukelić Ž., Clemmer D.E., Zamfir A.D. (2016). Electrospray Ionization Ion Mobility Mass Spectrometry of Human Brain Gangliosides. Anal. Chem..

[12] Ica R., Petrut A., Munteanu C., Sarbu M., Vukelić Ž., Petrica L., Zamfir A.D. (2020). Orbitrap mass spectrometry for monitoring the ganglioside pattern in human cerebellum development and aging. J. Mass Spectrom..

[13] Schnaar R.L., Kinoshita T., Varki A., Cummings R.D., Esko J.D., Freeze H.H., Stanley P., Bertozzi C.R., Hart G.W., Etzler M.E. (2017). Essentials of Glycobiology.

[14] Brandenburg K., Holst O. (2015). Glycolipids: Distribution and biological function. eLS.

[15] Telano L.N., Baker S. (2021). Physiology, cerebral spinal fluid. StatPearls, Treasure Island (FL).

[16] Füvesi J., Hanrieder J., Bencsik K., Rajda C., Kovacs S.K., Kaizer L., Beniczky S., Vecsei L., Bergquist J. (2012). Proteomic Analysis of Cerebrospinal Fluid in a Fulminant Case of Multiple Sclerosis. Int. J. Mol. Sci..

[17] Sasso B.L., Agnello L., Bivona G., Bellia C., Ciaccio M. (2019). Cerebrospinal Fluid Analysis in Multiple Sclerosis Diagnosis: An Update. Medicina.

[18] Ruprecht K., Tumani H. (2016). Cerebrospinal fluid diagnostics in multiple sclerosis. Der Nervenarzt.

[19] Pentsova E.I., Shah R., Tang J., Boire A., You D., Briggs S., Omuro A., Lin X., Fleisher M., Grommes C. (2016). Evaluating Cancer of the Central Nervous System Through Next-Generation Sequencing of Cerebrospinal Fluid. J. Clin. Oncol..

[20] Pan W., Gu W., Nagpal S., Gephart M.H., Quake S.R. (2015). Brain Tumor Mutations Detected in Cerebral Spinal Fluid. Clin. Chem..

[21] Lee J.S., Melisko M.E., Magbanua M.J.M., Kablanian A.T., Scott J.H., Rugo H.S., Park J.W. (2015). Detection of cerebrospinal fluid tumor cells and its clinical relevance in leptomeningeal metastasis of breast cancer. Breast Cancer Res. Treat..

[22] Powers C.J., Dickerson R., Zhang S.W., Rink C., Roy S., Sen C.K. (2016). Human cerebrospinal fluid microRNA: Temporal changes following subarachnoid hemorrhage. Physiol. Genom..

[23] Zetterberg H., Skillbäck T., Mattsson N., Trojanowski J.Q., Portelius E., Shaw L.M., Weiner M.W., Blennow K., Alzheimer’s Disease Neuroimaging Initiative (2016). Association of Cerebrospinal Fluid Neurofilament Light Concentration With Alzheimer Disease Progression. JAMA Neurol..

[24] Dayon L., Galindo A.N., Wojcik J., Cominetti O., Corthésy J., Oikonomidi A., Henry H., Kussmann M., Migliavacca E., Severin I. (2018). Alzheimer disease pathology and the cerebrospinal fluid proteome. Alzheimer’s Res. Ther..

[25] Schindler S.E., Li Y., Todd K.W., Herries E.M., Henson R.L., Gray J.D., Wang G., Graham D.L., Shaw L.M., Trojanowski J.Q. (2019). Emerging cerebrospinal fluid biomarkers in autosomal dominant Alzheimer’s disease. Alzheimer’s Dement..

[26] Nissen M.S., Nilsson A.C., Forsberg J., Milthers J., Wirenfeldt M., Bonde C., Byg K.-E., Ellingsen T., Blaabjerg M. (2019). Use of Cerebrospinal Fluid Biomarkers in Diagnosis and Monitoring of Rheumatoid Meningitis. Front. Neurol..

[27] Dittrich T.D., Marsch S., Egli A., Rüegg S., De Marchis G.M., Tschudin-Sutter S., Sutter R. (2020). Predictors of infectious meningitis or encephalitis: The yield of cerebrospinal fluid in a cross-sectional study. BMC Infect. Dis..

[28] Blinder T., Lewerenz J. (2019). Cerebrospinal Fluid Findings in Patients With Autoimmune Encephalitis—A Systematic Analysis. Front. Neurol..

[29] Doljanski F., Kapeller M. (1976). Cell surface shedding—The phenomenon and its possible significance. J. Theor. Biol..

[30] Milhorat T., Hammock K., Wood J.H. (1983). Cerebrospinal fluid as reflection of internal milieu of brain. Neurobiology of Cerebrospinal Fluid.

[31] Ladisch S., Chang F., Li R., Cogen P., Johnson D. (1997). Detection of medulloblastoma and astrocytoma-associated ganglioside GD3 in cerebrospinal fluid. Cancer Lett..

[32] Nordin V., Lekman A., Johansson M., Fredman P., Gillberg C. (2008). Gangliosides in cerebrospinal fluid in children with autism spectrum disorders. Dev. Med. Child Neurol..

[33] Ledeen R.W., Yu R.K., Volk B.W., Aronson S.M. (1972). Gangliosides of CSF and plasma: Their relation to the nervous system. Sphingolipids, Sphingolipidoses and Allied Disorders.

[34] Avrova N.F., Vlasova Y.A. (2018). Brain Gangliosides and Their Function as Natural Adaptogenes. Evolutionary Physiology and Biochemistry-Advances and Perspectives.

[35] Bohrer B.C., Merenbloom S.I., Koeniger S.L., Hilderbrand A.E., Clemmer D.E. (2008). Biomolecule Analysis by Ion Mobility Spectrometry. Annu. Rev. Anal. Chem..

[36] Kanu A.B., Dwivedi P., Tam M., Matz L., Hill H.H. (2008). Ion mobility-mass spectrometry. Biol. Mass Spectrom..

[37] Huang Y., Gelb A., Dodds E. (2014). Carbohydrate and Glycoconjugate Analysis by Ion Mobility Mass Spectrometry: Opportunities and Challenges. Curr. Metabolomics.

[38] Stow S.M., Lareau N.M., Hines K.M., McNees C.R., Goodwin C.R., Bachmann B.O., Mclean J.A., Havlíček V., Spížek J. (2014). Structural separations for natural product characterization by ion mobility–mass spectrometry fundamental theory to emerging applications. Natural Products Analysis: Instrumentation, Methods, and Applications.

[39] Jackson S.N., Ugarov M., Egan T., Post J.D., Langlais D., Schultz J.A., Woods A.S. (2007). MALDI-ion mobility-TOFMS imaging of lipids in rat brain tissue. Biol. Mass Spectrom..

[40] Jackson S.N., Colsch B., Egan T., Lewis E.K., Schultz J.A., Woods A.S. (2010). Gangliosides’ analysis by MALDI-ion mobility MS. Analyst.

[41] Wojcik R., Webb I.K., Deng L., Garimella S.V.B., Prost S.A., Ibrahim Y.M., Baker E.S., Smith R.D. (2017). Lipid and Glycolipid Isomer Analyses Using Ultra-High Resolution Ion Mobility Spectrometry Separations. Int. J. Mol. Sci..

[42] Sarbu M., Vukelić Ž., Clemmer D.E., Zamfir A.D. (2017). Electrospray ionization ion mobility mass spectrometry provides novel insights into the pattern and activity of fetal hippocampus gangliosides. Biochimie.

[43] Sarbu M., Vukelić Ž., Clemmer D.E., Zamfir A.D. (2018). Ion mobility mass spectrometry provides novel insights into the expression and structure of gangliosides in the normal adult human hippocampus. Analyst.

[44] Sarbu M., Clemmer D.E., Zamfir A.D. (2020). Ion mobility mass spectrometry of human melanoma gangliosides. Biochimie.

[45] Sarbu M., Petrica L., Clemmer D.E., Vukelić Ž., Zamfir A.D. (2021). Gangliosides of Human Glioblastoma Multiforme: A Comprehensive Mapping and Structural Analysis by Ion Mobility Tandem Mass Spectrometry. J. Am. Soc. Mass Spectrom..

[46] Sarbu M., Raab S., Henderson L., Fabris D., Vukelić Ž., Clemmer D.E., Zamfir A.D. (2020). Cerebrospinal fluid: Profiling and fragmentation of gangliosides by ion mobility mass spectrometry. Biochimie.

[47] Kusunoki S., Chiba A., Kon K., Ando S., Arisawa K., Tate A., Kanazawa I. (1994). N-acetylgalactosaminyl GD1a is a target molecule for serum antibody in Guillain-Barré syndrome. Ann. Neurol..

[48] Kaida K., Kusunoki S., Kamakura K., Motoyoshi K., Kanazawa I. (2003). GalNAc-GD1a in human peripheral nerve: Target sites of anti-ganglioside antibody. Neurology.

[49] Kaida K., Sonoo M., Ogawa G., Kamakura K., Ueda-Sada M., Arita M., Motoyoshi K., Kusunoki S. (2008). GM1/GalNAc-GD1a complex: A target for pure motor Guillain-Barre syndrome. Neurology.

[50] Kaida K., Kusunoki S., Kamakura K., Motoyoshi K., Kanazawa I. (2000). Guillain-Barré syndrome with antibody to a ganglioside, N-acetylgalactosaminyl GD1a. Brain.

[51] Koga M., Gilbert M., Takahashi M., Li J., Hirata K., Kanda T., Yuki N. (2012). GQ1b-seronegative Fisher syndrome: Clinical features and new serological markers. J. Neurol..

[52] Yoshino H., Ariga T., Suzuki A., Yu R.K., Miyatake T. (2008). Identification of gangliosides recognized by IgG anti-GalNAc-GD1a antibodies in bovine spinal motor neurons and motor nerves. Brain Res..

[53] Yoshino H. (1997). Distribution of gangliosides in the nervous tissues recognized by axonal form of Guillain-Barré syndrome. Neuroimmunology.

[54] Zamfir A., Vukelić Ž., Bindila L., Peter-Katalinić J., Almeida R., Sterling A., Allen M. (2004). Fully-automated chip-based nanoelectrospray tandem mass spectrometry of gangliosides from human cerebellum. J. Am. Soc. Mass Spectrom..

[55] Svennerholm L., Fredman P. (1980). A procedure for the quantitative isolation of brain gangliosides. Biochim. Biophys. Acta Lipids Lipid Metab..

[56] Vukelic Z., Metelmann W., Müthing J., Kos M., Peter-Katalinic J. (2001). Anencephaly: Structural Characterization of Gangliosides in Defined Brain Regions. Biol. Chem..

[57] Svennerholm L. (1980). Ganglioside designation. Adv. Exp. Med. Biol..

[58] Chester M.A. (1998). IUPAC-IUB joint commission on biochemical nomenclature (JCBN). Eur. J. Biochem..

[59] Domon B., Costello C.E. (1988). A systematic nomenclature for carbohydrate fragmentations in FAB-MS/MS spectra of glycoconjugates. Glycoconj. J..

[60] Costello C.E., Juhasz P., Perreault H. (1994). Chapter 4 New mass spectral approaches to ganglioside structure determinations. Prog. Brain Res..

[61] Ann Q., Adams J. (1992). Structure determination of ceramides and neutral glycosphingolipids by collisional activation of [M + Li]^+^ ions. J. Am. Soc. Mass Spectrom..

[62] Serb A., Schiopu C., Flangea C., Sisu E., Zamfir A.D. (2009). Top-down glycolipidomics: Fragmentation analysis of ganglioside oligosaccharide core and ceramide moiety by chip-nanoelectrospray collision-induced dissociation MS^2^-MS^6^. J. Mass Spectrom..

[63] Ghiulai R.M., Sarbu M., Vukelić Ž., Ilie C., Zamfir A.D. (2014). Early stage fetal neocortex exhibits a complex ganglioside profile as revealed by high resolution tandem mass spectrometry. Glycoconj. J..

[64] Schiopu C., Vukelić Ž., Capitan F., Kalanj-Bognar S., Sisu E., Zamfir A.D. (2012). Chip-nanoelectrospray quadrupole time-of-flight tandem mass spectrometry of meningioma gangliosides: A preliminary study. Electrophoresis.

[65] Robu A.C., Vukelić Ž., Schiopu C., Capitan F., Zamfir A.D. (2016). Mass spectrometry of gangliosides in extracranial tumors: Application to adrenal neuroblastoma. Anal. Biochem..

